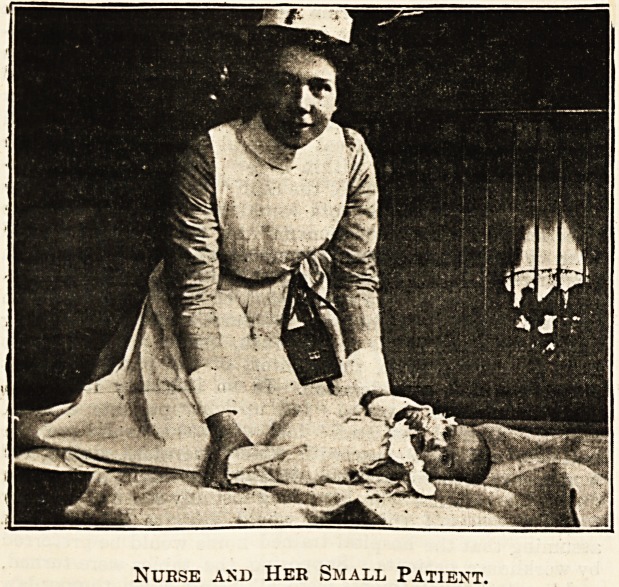# Nursing Section

**Published:** 1903-12-26

**Authors:** 


### The Hospital.

### Nursing Section.

Contributions for this Section of "THE HOSPITAL" should be addressed to the EDITOR, "THE HOSPITAL" NURSING SECTION, 28 & 29 Southampton Street, Strand, London, W.C

NO. 900.—VOL. XXXV. *SATURDAY, DECEMBER* 26, 1903.

### Notes on News from the Nursing World.

### OUR CHRISTMAS DISTRIBUTION OF CLOTHING.

THE garments sent to us for distribution at Christmas, between 200 and 300 in number, and forwarded to a number of hospitals, are acknowledged in another column. The letters of the various matrons will, we are sure, be read with great interest and intense satisfaction by all our kind-hearted contributors, who will learn, therefrom, how warmly their efforts are appreciated not only by the patients but also by those who are responsible for the charge of them. In addition to articles already acknowledged, we have received parcels from Nurse Ruel, Ryde, Isle of Wight; Nurse Hitchcock; and a box of toys from Mrs. Tennyson Harvey, Wolseley House, 20 St. James's Road, Hastings, which we have forwarded to the Victoria Hospital for Children, Chelsea.

### PRINCESS LOUISE AT GLASGOW.

FOLLOWING her visit to Edinburgh, the Princess Louise, Duchess of Argyll, travelled to Glasgow last Thursday in order to preside at the eleventh annual meeting of the Glasgow and West of Scotland Co-operation of Trained Nurses. The Princess on entering the room in which the meeting was held at the Central Station Hotel, was presented by Miss Newman with a bouquet. Having formally moved the adoption of the report, to which we referred last week, her Royal Highness was succeeded by the Lord Provost, who, in the course of a eulogistic speech on the Co-operation, said that they would always in the future history of the movement associate the name of the Princess and her gracious presence with a meeting which he regarded as full of hope and interest for the society. Subsequently the Duke of Argyll, responding to a vote of thanks to the Princess for her presence, declared that she thought it a great privilege to be able to take part in the work.

### THE IMPERIAL MILITARY NURSING SERVICE.

WE are officially informed that the following appointments have been made to Queen Alexandra's Imperial Military Nursing Service:—Matron: Miss E. A. Wilkinson, posted to Woolwich. Sisters: Miss F. M. Hodgins, Miss E. H. Hordley, and Miss M. Steenson, all stationed in South Africa. Staff Nurses: Miss A. L. Walker, stationed in South Africa; Miss M. L. Harris, stationed at Portsmouth; Miss S. K. Bills and Miss A. E. Fitzgerald, posted to Netley; and Miss B. N. Daker, posted to the Cambridge Hospital, Aldershot. The following ladies have been confirmed in their appointments, their period of provisional service having expired:—Sisters: E. C. Cheetham, S. Lamming, G. E. Larner, L. M. Lyall, E. C. Stewart, and I. G. Willetts. Staff Nurses: E. M. Bickerdike, M. M. Blakely, M. M. Bond, A. F. Byers, A. Fitzgerald, E. C. Humphreys, M. Kendall; M. Pedler, E. M. Pettle, M. L. Potter, L. A. Rideout, M. M. Tunley, K. Ward, and A. A. Wilson. Sisters G. A. Magill and E. C. Stewart have embarked in the s.s. *Plassy* for Indian troopship duty.

### THE SECRETARY FOR WAR AND POOR-LAW NURSES.

THE Clerk to the King's Norton Board of Guardians has been the means of eliciting an important declaration from the Secretary of State for War. Miss F. Savage, a nurse who had been trained at Selly Oak Infirmary, applied for an appointment in Queen Alexandra's Imperial Military Nursing Service, and received a letter in which it was stated that her training had not been carried out at an institution, the course of which was recognised as qualifying for the Imperial Military Nursing Service. Acting for the King's Norton Guardians, who are responsible for the Selly Oak Infirmary, the Clerk wrote to the War Office, and pointed out that the beds at the infirmary number 250, that there is a resident medical officer and a highly-trained matron, and that the probationers receive a thorough training for a period of three years in medical, midwifery, and general nursing, obtaining a certificate only after an examination conducted by one of the leading medical practitioners in Birmingham. After some delay the War Office replied, expressing regret that the Clerk "should have been given to understand that a nurse trained in the Selly Oak Infirmary was not eligible for an appointment in Queen Alexandra's Imperial Military Nursing Service," and stating "that there is no intention to exclude nurses otherwise deemed eligible who have received their training in large Poor-law Infirmaries under the Local Government Board." Poor-law nurses who have been trained at institutions fulfilling the same conditions as Selly Oak Infirmary, have thus an authoritative assurance from the fountain head that they will be considered qualified for service in the Army.

### DISCHARGE OF A NURSE AT BURY.

AT the fortnightly meeting of the Bury Board of Guardians last week the visiting committee reported that the workhouse master had notified that through the carelessness of a nurse the body of a female inmate who had died had wrongly been taken out of the workhouse for burial, and had to be brought back. The Committee advised that the nurse should be reprimanded, requested her to send an apology to the Guardians, and to pay the undertaker's expenses

At the meeting it was stated that the nurse had promised to conform to the wishes of the Committee, but had subsequently written a letter in which she said that although the mistake had not been hers, and the persons had not been brought face to face to prove it, she had decided to send in her resignation as assistant nurse. The chairman characterised the letter as an act of defiance to the Board, and it was determined that the resignation of the nurse should not be accepted, but that she should be discharged.

### HACKNEY INFIRMARY SCANDAL.

Upon inquiry at the Hackney Workhouse Infirmary, our representative found that neither the matron nor the clerk to the Guardians was willing to say anything respecting the very serious charges which have been made against a member of the Board by the nurses. The clerk handed our representative a copy of a local paper, but remarked that he would not vouch for the accuracy of the report of the proceedings. We do not find that the report contains any statements which it is either necessary or desirable to recapitulate at this period. The main points were given in our columns last week, and as the matter has very wisely been remitted to the Local Government Board to deal with, comment would be out of place. With reference, however, to the fact that the alleged brawling which engaged the attention of the Hackney Board of Guardians for three hours, occurred on Sunday, we suggest that Sunday is not a suitable day for Guardians to examine lifts.

### A JOKE OUT OF PLACE.

In the course of the hearing last week of a charge in the Rotherham Borough Court against a miner accused of using violent, obscene, and threatening language to a probationer in the Rotherham Workhouse Infirmary, an incident occurred which should act as a warning to young nurses to exercise self-restraint. The probationer, who gave evidence against the prisoner, which was to the effect that he charged her with having administered poison to him instead of his proper medicine, threatened to strike her, and was very abusive, was asked by him if she did not say some weeks ago that she would like to prepare him for the dead-house. Her reply was "I did so in a joke." There is no excuse for the misconduct of the man, who was properly convicted and sent to prison for seven days; but it was very wrong and very silly of the probationer to indulge in such a grim joke. We do not wonder that the patient in his ignorance took it seriously, and perhaps believed that he had intentionally been given poison to hasten his end. Sick people naturally object to be told, even by way of a joke, that their death would be welcomed.

### THE NURSE AND THE HOSPITAL COMMITTEE.

Judgment was given the other day in a Welsh County Court for £3 7s. 3d. against Miss Blanche Bradbury, a charge nurse at Exton Hospital, near Middlesbrough. The claim was made by the committee of Porth Cottage Hospital, Glamorganshire, who advertised for a matron at a salary of £40. It was stated in the advertisement that she must be thoroughly qualified to train probationers, well up in surgical and gynæcological work, and must enter upon her duties at the end of last July. Miss Bradbury was one of the selected candidates, and at the request of the committee she travelled to Wales in order to be interviewed. She was appointed, but discovering subsequently that the hospital was not what she expected, she sent in her resignation within twelve hours of her appointment, or almost two months before she was required to take up her duties. The secretary wrote her in reply that her resignation could only be accepted on condition that she paid a month's salary in "lieu of notice." This she refused to do, and eventually she received a summons for £5 10s. At the hearing the committee were awarded not £5 10s. but £3 7s. 3d., the actual amount spent by the Porth Cottage Hospital Committee in getting down two other candidates from London. It was agreed that there should be no solicitor's fees on either side. There is no doubt that the committee had law on their side, but if, as we are informed, there were three other candidates available, their action in suing a nurse at present earning only a salary of £30 a year under the circumstances described, seems rather regrettable.

### A QUESTION OF DISCIPLINE AT TRIM.

The Irish Local Government, having decided that Miss Margaret J. Sheridan, in declining to take charge of the consumption patients in the Trim Union Infirmary at night, had no adequate grounds for not discharging this part of her duty, and in these circumstances refused to remove her suspension, it remained, of course, for the Guardians to settle the matter. At their subsequent meeting they resolved: "That Miss Sheridan be called upon to apologise for not obeying the Board's order of October 17th, and that she refund the difference between her salary and the salary of temporary trained nurse employed to do her duty. Nurse Sheridan to be reinstated as nurse if she carries out that order. A week to be allowed her to consider her decision." The week expired on Friday last, and we understand that Miss Sheridan, whom the Guardians all describe as an excellent nurse, accepted the conditions of the Guardians and has therefore been reinstated. If the difference in her salary and that of the temporary trained nurse employed to do her duty is, as we are informed, £11, she has had to pay dearly for a breach of duty. Such, however, it was, and a breach of duty cannot be overlooked without disastrous results to the discipline of an institution. But we hope that the Trim Guardians will now endeavour to comply with the suggestion that the yards which the night nurse has to traverse between the new and the old wards in the infirmary should be covered.

### TRAINED NURSES AND DOMESTIC SERVANTS.

There are still Poor-law guardians who desire to treat trained nurses as if they were domestic servants. This explains a scene which took place at the last meeting of the Colchester Board, when a long and angry discussion arose on a motion for the appointment of a woman to do the domestic work in the nurses' home. Several guardians expressed an opinion that the nurses should "clean up" for themselves, in spite of the fact that it was shown they are fully engaged with their professional duties in the workhouse infirmary. These guardians appear to be the same persons who on a former occasion opposed the opening of a nurses' home. In the end their efforts failed, but the principle that a trained nurse should in effect be treated as a domestic servant was supported by nine against ten. We rejoice to learn that the three ladies voted with the majority.

### WORKHOUSE MASTERS AND THE SUPERINTENDENT NURSE.

At the recent meeting of the National Association of Workhouse Masters and Matrons at Birmingham, there was a good deal of criticism of the speeches at the conference convened by the Hospitals Association; but the practical outcome of a long discussion on the nursing problem was a unanimous decision to support the resolution carried at the annual meeting of the Council of the Poor-Law Unions' Association so far as relates to the order of precedence in the sick wards, and generally as to the suggested relationships between the offices of master and matron and master and superintendent nurse. The "creation of the position of superintendent nurse" was denounced by one of the masters as "a grave error of judgment." Her creation, remark, never have been necessary except for the existence of the untrained matron, whom alone we desire her to supersede.

### THE AMUSEMENTS OF HOSPITAL NURSES.

There has been a discussion by the Stockport Town Council on the subject of the amusements of hospital nurses. The members of the Sanitary Committee who had visited the Borough Hospital reported that there was "an urgent need for a new piano for the nurses," whereupon one member of the Council said that the nurses must have got very musical, for he noticed that there had been donations for a gramaphone; while another observed that if they were able to use both gramaphone and piano they could not have enough work to do. Dr. Smeeth rejoined that he knew the nurses were hard worked, and contended that in their off-duty time they ought therefore to have as much recreation as possible. This reasonable view of the matter was followed by a hint from the chairman of the Sanitary Committee that the example of ten members of the Council who had subscribed a guinea each towards the purchase of an instrument should be emulated by the remainder. We hope that the "urgent need" will be met in the suitable manner suggested by the chairman of the Sanitary Committee.

### A GOOD START AT SHEFFIELD.

A suitable and commodious Nurses' Home, has just been acquired by the Sheffield Queen Victoria's District Nursing Association, and the work of the organisation itself has been simultaneously inaugurated. An excellent start has been made with four nurses, the estimated cost of each being £100 a year, and it is hoped that the staff will be speedily increased to eight, exclusive of the superintendent. The expense of furnishing the Home, which it is intended to extend when the additional nurses are engaged, is about £500. The four nurses, in the first week in November, paid 150 visits, and in the first week in December the number had increased to 160. The need of the Association has thus been clearly shown, and we trust that it will receive adequate financial support.

### CHICAGO VISITING NURSES AND THE PREVENTION OF TUBERCULOSIS.

Under the auspices of an influential advisory committee an organised effort is being made in Chicago to carry out measures for the prevention of tuberculosis. An office connected with those of the Visiting Nurse Association has been rented, and there is a doctor in attendance every afternoon to see any patients sent him by the visiting nurses, or charity workers in the city. The visiting nurses are supplied with a table of instructions for the care of tuberculous patients, and with cards of record of "history" of each patient. The movement was started at the beginning of the year by the directors of the Visiting Nurse Association, and the active co-operation of the nurses appears to have had such practical results that it is proposed to enlarge the work. The qualifications of a careful, well-informed and observant trained nurse to assist in the task of finding out the districts where tuberculosis is most prevalent, and to ascertain the sanitary, or insanitary, conditions in force, cannot be doubted.

### OPERATIC PERFORMANCES AND DISTRICT NURSING.

Last week the Portsmouth Amateur Operatic Society gave a series of performances of the "Gondoliers," in the new Theatre Royal, Landport, in aid of the Portsmouth Victoria Association for Nursing the Sick Poor, by special request of the Mayor, Major J. E. Pink. The Association has been in existence since 1884, when three nurses took charge of the district. The staff now consists of a lady superintendent, four staff and 12 assistant nurses. The work is carried on so economically that the average cost of each visit is only 5½d., but in spite of this, the receipts fall a little short of the expenditure, whilst the mortgage on the Nurses' Home, in the High Street, is a serious matter. The Home is close to the Dockyard, and not the least interesting part of the nurses' duty lies amongst the sailors and marines.

### SOCIAL GATHERING IN SOMERSET.

The nurses of Taunton and neighbourhood met recently at the Grange, Kingston. Mr. L. Birkbeck, M.B., lectured on "Anatomy from a Nursing Point of View," and demonstrated his facts on a full-sized anatomical figure. There was on view a small collection of new nursing requisites, a number of pamphlets, and a microscope with physiological and pathological slides.

### SHORT ITEMS.

A nurse's home and bureau, on co-operative principles, is to be opened at 86 Lower Leeson Street, Dublin, on January 1st, 1904, with Miss Margaret E. MacDonnell—who was trained at Edinburgh Royal Infirmary, and was from 1892 to 1903 matron of the Travancore Station Hospital, India—as lady superintendent.

### The Nursing Outlook.

"From magnanimity, all fear above;
From nobler recompense, above applause,
Which owes to man's short outlook all its charm."

### NURSES AND THE NATIONAL PHYSIQUE.

There is no more marked want amongst our sick poor than greater knowledge of the importance of hygiene, and of how to secure cleanly and healthy conditions. And there is no more marked want in the nursing world than public impersonal feeling to mitigate the excessive absorption in pressing daily duties. These two things act and react as regards our national physique—a pressing question of the day.

Thanks mainly to the late Mr. Rathbone and to the Queen Victoria Jubilee Institute, we are ahead of all other nations in regard to district nursing; the new countries are following quickly in our steps however, more quickly than the towns of the Continent. As concerning the health of the people in their homes there can be no doubt that the district nurse is a most valuable adjunct; she teaches the poorest by example, by her own spotless dress, her own neat methods and careful use of appliances; and to low intelligences it is practice only appeals—theory is useless, and lectures on hygiene are not attended, or if attended are not understood. But when a poor woman sees a district nurse roll up her sleeves and plunge her hand and arms well in the water and use soap lavishly she gets a new idea of what washing the hands means. When she sees the difference in the appearance of a room that has been cleaned under the directions and with the help of a nurse; when she sees the difference in the appearance of the sick child whose bed and toilet have been attended to thoroughly, the vision remains. It cannot be too strongly insisted upon that the only way to teach hygiene to those who most need the lesson is by example, by appealing to the hand and eye. There is sitting just now at Whitehall a small committee of gentlemen to take evidence as to the supposed degeneration of our national physique, and so far they have heard only military men who no doubt advocated physical exercises and compulsory medical service. But this is horribly like the race Gulliver came across in his travels who began everything at the top—if they wanted to build a church they began with the steeple not with the foundations. If we want to improve the health of the people of England we must begin at the bottom; we must begin by teaching cleanliness and the proper feeding of infants and the care of little children to the dwellers in the slums. When a child is already stunted and diseased by improper feeding and foul air, it is useless to try and make a man of him by teaching him military drill. Here is an important national question in which the nurse should come forward and do service for the Empire—service as great as any soldier can render. For it is the district nurse and the school nurse who know best the need of health teaching, and the method of health teaching, and the conditions of life amongst the very poor. The tale the nurse could unfold to that committee of gentlemen—the tale of the puny infant reared on bread and beer, the tale of the little child forced unfed into our schools to do brain work for five hours a day, the tale of the crowded rooms, the verminous beds, the filthy clothing, and all the unhealthy details which go to rear millions of puny, pitiful men and women—that is the tale that only a woman can tell and only a nurse can understand. These are the foundations of the feebleness of our fighting force, these are the causes of our unemployed and unemployable, these are the reasons for our decline not only in physical but also in mental and moral health. You cannot rear a strong nation on a feeble foundation; and you cannot build a cathedral if you begin with the spire. If we had our nursing organisations in good working order, we should long before this have secured the presence of a woman on the Physical Degeneration Committee, and we should have had our district and school and out-patient nurses busily engaged in compiling facts which a central nursing body would tabulate and lay before the committee. But though in devotion and good work the English nurse is unsurpassed, she is not the equal of her American sister when it comes to a question of public spirit and organisation. We are too narrow in our views, too wedded to detail, too lacking in self-reliance. The health of the nation may secure the work of our hands, but not the collective wisdom of our heads. And so, once more, a great opportunity must pass, and as Mazzini said—"The cause of our failures is in ourselves: in our want of organisation, in our ceaseless distrust, in our miserable little vanities, in our absolute want of that spirit of discipline and order which alone can achieve great results."

Meanwhile in New York the school nurses working directly under the municipality have been increased to 35. The Visiting Nurses' Association of Chicago reports 15 nurses in active work, who brought to Dr. Lorenz a large number of patients who could have reached him in no other way. These nurses report that they supply soap, towels, and tooth-brushes to all children who cannot afford them. And the American Society of Superintendents has established a Teachers' Hospital Course, has sent representatives to Congress, have established an excellent journal, and planted and cultivated associated alumnæ; and it has lately appointed a standing committee to "watch public events as related to nursing, and to make the voice of the Society constantly heard, whether in criticism, in commendation, in warning, or in petition." Nurses in America are an effective public force; for the sake of our sickly, undersized children we could wish, at the present juncture, that English nurses had like influence and weight.

### Lectures on Ophthalmic Nursing.

By A. S. Cobbledick, M.D., B.S.Lond., Senior Clinical Assistant and late House-Surgeon and Registrar to the Royal Eye Hospital.

### LECTURE XXV.—DISEASES OF THE OPTIC NERVE.

*Optic Neuritis* (inflammation of the optic nerve).—This may appear alone or associated with retinitis. The latter class of case has already been dealt with. The term "optic neuritis" applies to an inflammation of any part of the nerve, and not only to those cases where the disc is affected and the trouble diagnosed by means of the ophthalmoscope (neuritis intraocularis). When the nerve is inflamed behind the eye-ball (neuritis retro-ocularis) the fundus may appear to be quite healthy, but the vision is very much impaired. It is not uncommon to meet with intraocular neuritis accidentally; for there may be no complaint of loss of vision even when a severe degree of inflammation is present. *Vice versâ* vision may be much impaired when ophthalmoscopically the appearances point to only a slight degree of neuritis. The chief cause of optic neuritis is some form of cerebral tumour, *e.g.* glioma, sarcoma, gumma, cyst, abscess, or tuberculous mass (the first and last of these are especially common forms of cerebral tumour in children). Other causes are extreme degrees of anæmia, *e.g.* chlorosis, leucocythemia, etc.; syphilis, quite apart from cerebral disease. More uncommon causes are myelitis (inflammation of the spinal cord), lead poisoning, and hydrocephalus.

The appearances noted at the optic disc are the only indications of this inflammatory process: these vary from a hyperæmia of the disc with an indistinct outline to complete obliteration of the disc's outline and obscuration of the blood vessels. Slight degrees are indicated by engorgment of the veins and by their continuity appearing to be broken at the margin of the disc by the surrounding swelling; the disc's outline is lost, and pointed woolly-looking processes project over the surrounding retina. In severe cases hæmorrhages appear, and the process by extending to the macula causes great impairment of vision.

*Treatment.*—The cause of the neuritis must be diagnosed and treated; no special treatment to the eyes has any effect on the neuritis.

*Neuritis Retro-ocularis.*—The acute form is characterised by a sudden onset, very marked impairment of vision and non-implication of the nerve at the disc, so that the fundus examined with the ophthalmoscope is found to be normal. It may be caused by hæmorrhage, injury, growth, over-fatigue with severe chill, and in certain nerve affections, *e.g.* multiple sclerosis. Besides loss of sight, the patient may complain of considerable pain in the head or eyes.

*Prognosis.*—In those cases where the causation has to be ascribed to severe chill the prognosis is almost always satisfactory, and the same may be said of hæmorrhage in the sheath of the nerve: the recovery may be slow and occupy some months. A chronic form of this affection is the blindness caused by toxic poisoning (*toxic amblyopia*). By far the most common poison is tobacco, *i.e.* used in excess. Other poisons which produce a similar condition are lead, arsenic, alcohol, iodoform, and other poisons.

*Tobacco Amblyopia* is met with in men about or beyond middle age, and is usually the result of excess in smoking or chewing the strongest brands. It is well to say usually because a few cases are met with where quite a moderate allowance has been the cause of the disease: in these cases it is well to inquire fully into any other possible cause, *e.g.* over-indulgence in alcohol, occupation, etc.

In hospital practice most of these tobacco cases are found to occur in men who lead irregular lives: over-indulgence in alcohol as well as tobacco, too little sleep, and an insufficiency of good nourishing food are the main factors in a case. Many cases can be diagnosed from the patients' facial appearance; the typical case exhibits a dull, listless eye, a muddy complexion, foul breath, in part due to bad teeth, which receive no attention; and a general appearance of loss of vascular tone.

*Symptoms.*—Both eyes are always affected, though one to a greater extent than the other. As long as the sight in one eye only is impaired there is not much complaint; but directly the vision in the other eye is affected then there is difficulty in following an occupation, and advice is sought. Amongst the most common complaints are that everything seems to be seen through a slight fog; that ordinary print appears to be lighter in colour, and hence more difficult to decipher; that small coloured objects cannot be detected; and that everyone's face appears to be pallid and unhealthy. These latter symptoms are due to a defect in the centre of the field of vision. This defect is called a *scotoma*, and it is characteristic of this affection that there is a central scotoma for red and green. Patients often state that their vision is much better at night (*nyctalopia*). Examined with the ophthalmoscope the fundus shows no gross change.

*Prognosis.*—Provided that the cause is *completely* removed, this is good, though it may take six or eight months to obtain a cure in severe cases. Most cases get worse after treatment is begun, before any improvement can be recorded. Untreated cases, or those in which the cause is not removed, usually end in optic atrophy and complete blindness.

*Treatment.*—Remove the cause, prohibit the use of alcohol, and map out a regular routine of meals, exercise, and sleep. The two most useful drugs are strychnine and potassium iodide; the former may be gradually increased—by one-minim gradations—from ♏ v. three times a day to ♏ xx. t.d. or even ♏ xxv t.d. with good results. Of late Mr. Doyne has advocated the use of retinal extract internally: this preparation has been named Optocene, and consists of an extract of animals' retinæ. Severe cases have been cured by Optocene in a much shorter time than by the usual methods.

*Optic Atrophy.*—This is a most serious affection, for it attacks both eyes and produces a gradual but certain loss of sight, through the atrophy of the terminals of the optic nerve in the retina, and consequent failure of their function, which is to conduct the light stimuli to the brain.

There are two forms, viz. inflammatory and non-inflammatory. The inflammatory consist of those cases where a previous neuritis has caused so much damage that atrophy results (post-neuritic atrophy), where an inflammation has been set up by injury to the nerve or compression of it in the orbit. The non-inflammatory (commonly called primary) are mostly associated with spinal or central nervous diseases, as in Tabes dorsalis, which is a degenerative disease of the spinal cord, general paralysis of the insane and disseminated sclerosis.

*Symptoms.*—In addition to progressive loss of vision there is a gradual contraction of the field of vision and colour blindness; blue is the last colour to be recognised. The ophthalmoscope shows a *white* very clearly cut disc and very small arteries; otherwise the fundus appears normal. The prognosis is bad and treatment is of but little avail.

### Our Christmas Distribution.

THE following letters have been received by us from the recipients of parcels of garments which we were able, through the thoughtfulness and generosity of our readers, to distribute:—

The Matron of St. Thomas's Hospital, Albert Embankment, S.E., writes:—"I beg to acknowledge with most grateful thanks the parcels of clothing sent to the patients by the proprietors and readers of THE HOSPITAL, which are very acceptable."

The Matron of the London Hospital, Whitechapel, E., writes:—"It is with much pleasure that I write to acknowledge the splendid parcel of warm clothing which has arrived safely at the hospital. I am sure that the articles will be much appreciated by the patients. We often have a difficulty in providing for our large numbers of inmates, but gifts like yours render the task a much easier one."

The Matron of the Metropolitan Hospital, Kingsland Road, N.E., writes:—"I wish to thank you and the readers of TH. HOSPITAL most heartily for the parcel of useful garments you have so kindly sent to us; they will be such a boon this winter, for the poverty among our poor patients is dreadful, and we are so thankful to have some warm garments to give to those who are convalescent and just leaving the comforts of the hospital for their wretched homes. Please convey our grateful thanks to the kind donors."

The Matron of Tottenham Hospital, N., writes:—"I acknowledge with warm thanks the kind gift of most useful clothing sent by the proprietors and readers of THE HOSPITAL for distribution to the patients. The two scarlet bed-jackets are so pretty that they will be kept for ward use. The other articles will be given away on Christmas Day."

The Secretary of the East London Hospital for Children and Dispensary for Women, Shadwell, E., writes:—"On behalf of the Board of Management I beg to thank you very sincerely for the welcome present of clothing you have been so kind as to forward on behalf of the proprietors and readers of THE HOSPITAL, and which is much appreciated."

The Lady Superintendent of the East End Mothers' Home, 394 and 396 Commercial Road, E., writes:—"Most grateful thanks for the very useful garments you have so kindly sent again this year for the patients of this home. They will indeed appreciate them, and I feel sure if the kind workers could see the grateful look upon the poor mother's face when she receives a warm garment either for herself or baby they would be more than repaid."

The Secretary of Charing Cross Hospital, W.C., writes:—"I am directed by the Board of Governors to convey to you their sincere thanks for the kind gift of clothes for the patients from the proprietors and readers of THE HOSPITAL."

The Lady Superintendent of the Middlesex Hospital, W., writes:—"I beg to thank the proprietors and readers of THE HOSPITAL for the articles kindly sent to the Christmas Tree for the patients of this hospital."

The Secretary of the Cancer Hospital, Fulham Road, S.W., writes:—"Would you kindly accept, and also convey to your readers, the sincere thanks of my committee for your very appreciable gift of clothing. It is our endeavour each year, on Christmas Day, to make a personal present to each patient; this year we were very anxious indeed about maintaining this custom, but your generosity has removed that load from our shoulders."

The Matron of Poplar Hospital for Accidents, East India Road, Poplar, E., writes:—"I desire to acknowledge, with very sincere and grateful thanks, the receipt of a parcel of clothing sent for our patients by the proprietors and readers of THE HOSPITAL. The garments are most acceptable, and they are so pretty as well as being so useful, and I am sure that those of our men who are fortunate enough to get the warm underclothing sent have reason to be most thankful to those kind friends who have given up so much of their time to this noble work."

The Matron of the London Temperance Hospital, Hampstead Road, N.W., writes:—"Allow me to thank you, and through you, the readers of THE HOSPITAL, who so kindly contributed to the bundle of warm garments which came for the patients yesterday. I hope to distribute them on the evening of the 28th, when we have our Christmas Tree, and can assure the kind givers of the very hearty appreciation of their gifts."

The Matron of the West Ham Hospital, Stratford, E., writes:—"Please accept my very sincere thanks for the parcel of most useful garments received from the proprietors and readers of your valuable paper, on behalf of our patients. This is the first year that my hospital has benefited by your generosity and I trust that it will not be the last. The garments sent have been of the greatest help in providing suitable presents for the men and women."

### Dot and Tiny.

### A TRUE INCIDENT.

THE two pets of our ward were Dot and Tiny. Dot had been brought in suffering from burns caused by a lamp exploding, and Tiny, who came in the week after, had a fractured arm. As soon as they were able to get up they became inseparable friends.

Tiny used to toddle up to Dot's cot with her dolly, and it was quite amusing to watch her efforts to keep Dot interested.

On "visiting days" Tiny would bring her daddy to Dot to share him with her, as her mother, who was a charwoman, could not leave her work, but on Christmas Day, to Dot's great delight, the mother came, and soon after Tiny's daddy arrived.

Of course he had to pay his usual call at Dot's cot, and then a strange thing happened. I saw in a moment that the man and woman were old friends; by a supreme effort the poor woman kept herself from falling and struggled to a seat. I went over to see if I could assist her, but she politely declined. The man bent over her and fanned her with his cap and seemed greatly affected. Soon after they left the ward together, after kissing the children.

To my surprise I received a letter from the woman a day or two later. She said that as I had been so kind to her little girl she would like to explain her strange conduct. Then she went on to say Tiny's father had been engaged to her years before, but they had quarrelled and parted, each marrying somebody else, and this had been the first meeting since the quarrel. They had both lost their partners in life, so were free at last to become united.

A few weeks after when they each claimed their little ones from us, sad as we were at losing two such darlings, it made us feel very happy to see such delight depicted on their two little faces when they heard that they were sisters, and were not going to be separated. It was a truly happy quartette that bade us good-bye that snowy January day.

### The Work of a Small Provincial Hospital.

BY A NURSE.

The Memorial Hospital at Kendal is a very good example of its kind. It is the only general hospital in the county of Westmorland; it serves a large country district as well as the busy and rapidly increasing little town,

It is in the uncomfortable stage of expansion, being neither a true cottage hospital, nor has it yet quite found its feet as a county hospital. In fact it is a building that has to be made to serve purposes quite different from those originally intended by its builders and donors.

It was originally erected in 1869 as a memorial to the late Mrs. Cropper, of Ellergreen. It was a purely cottage hospital of, I think, ten beds, with a working matron and one nurse. A children's ward was further added in 1878 together with more domestic accommodation, and the hospital now contains 25 beds.

The children's ward is the only one that can really be called in any way adequate, the others are much overcrowded, and two are cellar wards totally unfit for their purpose. The accommodation for the nurses and servants too is insufficient, and requires re-arrangement. All these drawbacks the committee fully realise, and they now have under consideration schemes for remedying matters, and making the hospital fully efficient and bringing it up to date in every way. They have made an excellent start by greatly improving the lavatory accommodation and rendering the building thoroughly sanitary—temporary measures greatly appreciated by the nursing staff.

The Exterior of Kendal Hospital.

### The Staff and Work.

A matron, two qualified staff nurses, and two probationers form the nursing staff, while the domestic side of the establishment is represented by two ward-housemaids and a cook. The cook and the senior housemaid have been in the service of the hospital, one eleven, the other twelve years. Porter there is none, but nurses and servants are all capable of stretcher-carrying, etc., and the convalescent Westmorlander forms a most teachable and handy ward orderly. The work done is good, the recovery record being excellent, the death-rate small. During the last six months three patients have died, all of whom were in a dying state when admitted. Since last December till the end of May the hospital has been constantly and uniformly full with a large percentage of cases requiring the administration of an anæsthetic. The average number of beds filled too is greatly on the increase, showing that the work of the hospital and its services are appreciated. A large number of eye cases—in proportion to the number of beds—are received and treated with most successful results.

### The Nursing.

Nursing in Westmorland is pleasant work: the Westmorlander is a cheery, high-couraged soul, who makes the best of illness or accident, and is not much troubled with "shock." He is always ready to render his nurses all the assistance in his power too; he will dust the ward, mind the hospital baby, fill hot-water bottles, or wash the hospital dog with the greatest of pleasure and good humour, and is always ready with some witty remark. His manners may perhaps, to the south countryman, seem a trifle abrupt on first acquaintance, but you will soon find the sterling worth of the man beneath, rugged like the crags he dwells amongst, but staunch and brave and true to the backbone, with a dry humour and pleasant wit withal, and his dialect and soft accent are most charming to listen to, though the former at times is a little difficult to understand.

### The Ambulance Brigade.

Kendal is also to be congratulated upon the possession of one of the most efficient ambulance brigades in the country. This brigade was only beaten in the recent competition for the championship shield by the London team, the Kendal men bearing off the Symons Eccles Cup, and taking the position of second in the country. Their work is admirable in every detail: first aid is well and efficiently rendered, transport is good, and in the wards their quiet and efficient help is a great assistance to the nurses.

The surgeon to the corps, who gives them their instruction, is to be congratulated on the smartness of his men, and on the good work they have done and are doing. Great credit, too, is due to their superintendent, a most smart officer, who, with Dr. Cockill, was largely instrumental in forming the corps four years ago, and who still works very hard in their interests.

The Kendal Memorial Hospital is up to date in one respect: it possesses an $x$-ray outfit with an extremely good coil. It also possesses an operating table of modern design. Aseptic surgery, of course, is an impossibility, but antiseptic principles are most carefully practised with excellent results, and the probationers get a good grounding in the elements of their work.

A Contrast.

Probationers are taken for one year, if after a trial month they afford satisfaction to the matron. Certificates, of course, are not issued, but if the probationer's work and conduct have been satisfactory, a testimonial is given to

Nurse and Her Small Patient.

her. She has, too, complete indoor uniform provided, the dresses even being made for her, and a salary of £10 per annum and laundry.

### TRAVEL NOTES AND QUERIES.

By our Travel Correspondent.

Genoa to Naples by Sea (E. C.).—Steamers make the passage every Saturday. Also frequently on Tuesday, but they do not guarantee Tuesdays. First class £2 10s. 5d., second £1 13s. 7d. It is often a very bad passage, and I should not recommend it. It is an Italian company, but Messrs. Cook, of Ludgate Circus, would take your passage for you.

### New Books on Nursing.

A Nurse's Handbook of Obstetrics for Use in Training Schools. By Joseph Brown Cooke, M.D. (Published by J. B. Lippincott Company, Philadelphia and London. 1903. 8vo. Pp. 391. 327 Illustrations. Price 9s. net.)

This volume is, so far as we are aware, at present unique of its kind, at any rate in this country, and we have derived pleasure from a perusal of its pages. The author seems to us to have been catering rather for the nurse, strictly so called, than for the midwife who conducts confinements on her own responsibility. We are quite at one with the author in his statement that the education and intelligence of the nurse are "the best and surest safeguards against insubordination and usurpation of authority." Books of this class, which are designed in part to prevent the nurse from using those highly technical medical works from which she can gain but doubtful benefit, are assuredly gaining ground in spite of the opposition of those who are so afraid of "over-educating our nurses." The book is profusely, one might say lavishly, illustrated, and this forms a marked feature of the work, the illustrations being, for the most part, models of clearness and simplicity. For a proper understanding of obstetrics copious diagrams and figures are a necessity, especially in a book designed for nurses who have not had the advantage of the previous scientific training enjoyed by the medical student. It is impossible for us to do more than make a very brief survey of the volume as a whole. There are, as would be expected, various points in which we in England differ from our American confrères, one or two of which we shall refer to in this notice. In the introduction the author points out the unique opportunities possessed by a trained obstetric nurse in imparting knowledge and advice to her own sex in regard to the care of the body during pregnancy and the requirements of the pregnant state, advice which the doctor is seldom asked to give, partly, no doubt, from motives of delicacy. Here, then, is a good opportunity for the skilled obstetric nurse to assist her sex. The planning of the book is on the whole to our liking, but we consider that the physiology of pregnancy and its symptoms and diagnosis are better treated in one chapter to be followed by a consideration of the disorders of pregnancy rather than preceded by it. Chapters which we consider to be specially well written are those dealing with the management of pregnancy, the conduct of "labour," the care of the normal infant and infant feeding. We are only selecting two or three minor points for criticism. In his account of the fœtal skull, the author describes the sagittal suture as starting at the base of the nose. This is contrary to the recognised teaching in this country. We are inclined to think figure 30, representing the striæ gravidarum, is calculated to give an erroneous idea of the size of the abdomen at term in natural pregnancy. A diagram, showing the natural rather than the unnatural in such a case, would surely be more serviceable. We can hardly favour as a routine practice the use of the hypodermic needle for injecting ergotin by the nurse, nor do we consider it to be part of her outfit. We, in this country, for the most part prefer as a routine practice the use of the left lateral position to the dorsal, and such an illustration as is shown in figure 56, to our mind involves an unnecessary exposure. Lastly, we do not consider that the exigencies of obstetrics require the nurse to be instructed in the administration of ether. A very full glossary of medical terms and a sufficient index complete a volume which we have pleasure in heartily recommending.

### Everybody's Opinion.

### DISCIPLINE AT CHARING CROSS HOSPITAL.

Miss J. McKerron, Matron of Chalmer's Hospital, Banff, N.B., writes: Allow me to confirm the statements made by sisters, past and present, of Charing Cross Hospital, regarding the former off-duty hours of the nursing staff. Neither on day nor night duty was a nurse allowed extension beyond the usual hours without the special permission of the lady superintendent, and I can testify from personal experience that that was not always granted. We certainly never considered that we were entitled to late leave once a week, but regarded it as a privilege to be granted at the discretion of the lady superintendent.

Miss Ada M. Smith, matron of the General Hospital, Tunbridge Wells, writes: As one who worked in Charing Cross Hospital for nine years—from 1894 till the present year—and in the interest of Charing Cross trained nurses, I feel justified in writing to ask that you will kindly insert the following facts in The Hospital, lest wrong impressions should have been gained from statements which have recently been published:—First, the term of training at Charing Cross has been three years, and the certificate given was for that time; secondly, neither sisters nor nurses have been allowed leave after 8 P.M. without the express permission of the lady superintendent.

"An Old Charing Cross Nurse" writes: May I be allowed to say a few words for Charing Cross Hospital? I was trained under the former matron, Miss Gordon, and always had fairness shown to me in every way. The matron seemed to know just how to deal with each individual nurse. It was the sisters whom I found hardest to get on with. They oftentimes gave a nurse under them a very hard time, but then one always felt, if things became too hard to bear, one would get justice from the matron, and disturbances were settled without public scandal. How it is now I do not know. I think it a pity that the weekly theatre leave has been taken away; after a hard day's work, rushing about the wards, it does not seem to me a nurse, especially a probationer, wants to be bothered with singing glees and such nonsense, but going to a theatre or concert she is taken completely away from her work, and at the same time enjoys and rests herself. This certainly does nobody any harm. I know that after going to a theatre I could always work with far greater will and pleasure. We were allowed theatre leave once a week, by asking the matron personally, and enjoyment such as one got helped to keep a nurse from becoming narrow-minded, which one is apt to get, always being in hospital.

### THE ANGLO-AMERICAN NURSING HOME AT ROME.

"Resident" in Rome writes: May I in connection with the Anglo-American Nursing Home lay before your readers a sad case which has come to my knowledge this week. A poor Englishwoman, of good position, was attacked by sudden dangerous illness, and taken to one of the hospitals on an ambulance, and is now in the common ward under most trying conditions, for which she has to pay two lire a day. She moans and says "if only things were different at the Anglo-American Nursing Home I should be there in one of the nice free beds, but as it is I could not go." Now is it not time that the public, or at least the subscribers, should demand an inquiry into the reason why those in dire need prefer to face the discomforts of the common hospitals in this city rather than take advantage of what has been provided for them by those who are better off? I enclose my card.

Miss Agnes Bulwer, Life Member of the General Committee of the Anglo-American Nursing Home, one of the dissentient minority, writes: I am delighted to learn from The Hospital of December 12, 1903, how deep an interest Sir Rennell Rodd, Secretary of the British Embassy, takes in the affairs of the Anglo-American Nursing Home at Rome; but as one of those who worked hardest for its establishment, who served on its managing committee for over a year—who only then retired because an inquiry into alleged mismanagement was not instituted—and who has been an interested reader, though not a writer, of the various letters lately published in The Hospital, I should like to draw Sir Rennell Rodd's attention as well as that of the managing committee to the issue of the controversy only just closed between a well-informed "dissentient minority" of the governing body of St. Bartholomew's Hospital, London, and the self-satisfied majority whose action has been condemned by press and public, while the minority have been upheld in their intentions. *Verb. sap.*

### POOR-LAW VACANCIES AND GENERAL HOSPITAL-TRAINED NURSES.

"Margaret" writes: I want to thank you for the article on "The Poor-law Nurse" in last week's Hospital. It is very good of you to find time to think of us at this busy season. The writer understands our position so well, and puts into words just what we Poor-law nurses feel. Many of us are watching the correspondence in your columns with keen interest, and hoping that Miss Twining and some of our guardians and matrons will be able to do something for us.

"H. B." writes: I think that "One of the Staff" displays uncalled for discourtesy in her reply to "Four Midland Nurses" and herself shows some ignorance of facts in her contradiction of their statement as to the testing of urine, etc., not being generally taught to nurses in hospital. Having received my training in an infirmary and in hospital, I can speak from experience of both trainings. In the infirmary the testing of urine and all the dressings were done by the nurses; in the hospital—a large one—these things were done by the clerks and dressers. In small general hospitals these duties may fall to the nurses' share, in the great hospitals, excepting the giving of hypodermics, they certainly do not. I remember perfectly well coming across a clever staff nurse of seven years' hospital experience who had never learnt urine testing, and who was taken aback at being expected to do it on taking up a new appointment. I beg to differ again with "One of the Staff" as to the likelihood of the Poor-law nurse being at sea if placed in a general hospital. I know at this moment of a Poor-law nurse who has shown so much knowledge and skill in the wards of a great general hospital that she has been made a sister within a few months of her admission to its wards. "One of the Staff" appears to insinuate that the workhouse patients would be nursed with more "tenderness" and "refinement" by the hospital-trained than by the infirmary-trained nurse. On thinking over both trainings I should certainly say that infirmary training is more conducive to the cultivation of these qualities than hospital training. In hospital the nurse is for ever working against time and in the general rush, bustle, and anxiety to keep the ward smart, the comfort of the patients is not always the first consideration. In an infirmary it is; the nurse has leisure there to "fad up" her patients a little, and if "One of the Staff" had witnessed, as I have, the patience and gentleness with which querulous, doting old women are treated, and the delicacy shown in performing duties for patients who have long lost any sense of decency for themselves, I think she would hesitate before again assuming that the hospital-trained nurse would be preferred by workhouse patients. Supposing the tables were turned, and hospital appointments were all being given to poor-law nurses, I should say, judging by the tone of her letter, that "One of the Staff" would be one of the first to cry out at the injustice of it. She should, therefore, try to forgive "Four Midland Nurses" and their followers for venturing to air what is after all a very real grievance.

### NURSES AS STEWARDESSES.

"Nautical" writes: Unless "M. W." knows anyone who has interest in any of the large liners I expect she will have great difficulty in obtaining the post of stewardess. My sisters and self were successful simply through having so many influential friends both in the Navy and Merchant Service. The only thing I can advise "M. W." is to go to the shipping agents and ask them to enter her name on their list. I should say one needed to be strong and in good health to take up the duties of a stewardess. To some people the extreme change of climate would be very trying, also the constant running up and down the companion. But as far as the work is concerned it is not hard.

"Disappointed" writes: At the termination of a long holiday, necessitated by a slight breakdown in health, I sought, by a doctor's advice, some occupation out of England. Seeing an advertisement in The Hospital for "lady stewardesses, hospital trained, wanted on a leading steamship line," I applied and was selected an eligible candidate. A list of the duties were given, none of which were menial, and the salary was at the rate of £36 per annum. The duties included taking the head of the children's mess, and attending to ladies indisposed in their cabins, etc., the yeomen doing all arduous duties, such as carrying water. I found, on arrival at the company's office, that another fully-certificated nurse was coming on the same boat as assistant-nurse-stewardess, and we were instructed by the manager to take all our orders from the purser. For the first three nights we shared a comfortable cabin, but on arrival at Lisbon the purser informed us that we must try and find sleeping accommodation in the ladies' waiting-room off the lavatories and bath-rooms, an extremely objectionable apartment, and to add to our discomfort, a black woman, nursemaid to a small passenger, was given a shake-down on the seats where we were trying to find some rest. The company had never taken ladies as nurse stewardesses before, and there seems some unwritten but understood law that the stewardesses, although they may be educated women, are not allowed on deck or to associate with the passengers in any way. The treatment given was exactly the same as to an uneducated woman of the domestic class, minus hospital training. It is almost needless to add that the experiment is one we should be sorry to repeat, and I only hope that any nurse who is tempted to travel in the same capacity will first ascertain if she is to be treated as a domestic or as a ship's officer, for on such a standing only can the arrangement produce comfort and position sufficient to attract educated women to take up the profession of trained nurse on board ship.

TRAINING UNDER THE ROYAL ARMY MEDICAL CORPS.

"Trained Male Nurse" writes: I was not surprised to see that some of the male nurses at the National Hospital should attempt to refute my contention that the best general training for a male nurse can be obtained in the Royal Army Medical Corps. But I did not claim that the training in the Royal Army Medical Corps was complete. Why now, after nearly seventeen years' experience, I am intensely disappointed if I do not pick up some new wrinkle every case I go to, and I should be delighted (if I could afford the time, to go through a course at the National, especially for massage and the application of electricity), but to assert that therefore the training at the National Hospital is the best, is as if a person should claim that a maternity hospital was the best place in the world for a lady to train at, to become a nurse, because nurses who have had years of training at St. George's, Guy's, etc., are glad to go through a further course at Queen Charlotte's, etc. Only a small minority of the men joining the corps are found to have a vocation for nursing, and a great deal will depend upon a man's luck, and what hospital a man is sent to after his first preliminary training at the Aldershot training school, and under what medical officers he has to do duty. Some doctors in charge of hospitals are great on drill, and will have several parades a day. Others will keep the orderlies severely under the thumb of the nursing sisters, whilst others again recognise how valuable really good male nurses are in the service, and when they find a man who likes the work give him all the facilities and encouragement possible. I had the good fortune to serve under such an officer, and in two years attended just two parades. I was employed as special orderly, *i.e.* had charge of not more than one case at a time, and that a case requiring special and careful nursing. At other times I was sent to nurse sick officers at their own homes. I then got what has been of great value to me, introductions and recommendations and also a knowledge of manners and customs in the houses of well-to-do people. I think it might be pressed upon the authorities that if they would uniformly give more encouragement, facilities, and promotion for nursing ability they would find no lack of skilful male nurses who in a campaign could be of incalculable value. In conclusion, I hope no one connected with the National Hospital will think I am trying to disparage their training. Far from it, but I must say that cases of paralysis are by no means the only cases that male nurses are called upon to attend. My present case is Bright's disease, my last pneumonia, before that renal calculus, and so on, and I am afraid I should not have given very great satisfaction if I had had training only in the management of nerve diseases.

### Novelties for Nurses.

By Our Shopping Correspondent.

THE WARD CHRISTMAS TREE.

Few children who have to spend Christmas in the hospital fail to receive a pretty gift. Yet this custom frequently entails an expenditure all too heavy for the slender means of sisters and nurses, upon whom the burden often falls. Those who spend most of their time within the hospital have few opportunities to make the most of the means at command, and buy from the nearest and often most expensive toy vendor. I think that readers may be glad to know that the T. B. L. Novelty House, Savoy Corner, Thames Embankment, supply a great variety of fascinating toys of the cheapest description, which are just as enticing to small people as the more costly trifles. They also provide nice little books of pictures to paint, the copies being produced in simply laid on, bright colours. The Novelty House also have a large assortment of Christmas and New Year's cards.

### Appointments.

**[No charge is made for announcements under this head, and we are always glad to receive, and publish, appointments. The information to insure accuracy should be sent from the nurses themselves, and we cannot undertake to correct official announcements which may happen to be inaccurate. It is essential that in all cases the school of training should be given.]**

Leicestershire Isolation Hospital, Syston.—Miss C. Shields has been appointed matron. She was trained at St. George's Hospital, London, and the South-Eastern Hospital, New Cross. She has since been doing private nursing for the Institution of Trained Nurses at Leicester.

### Presentations.

Edinburgh City Hospital.—Miss E. C. Sandford has been presented, upon her retirement from the post of lady superintendent of the City Hospital, Edinburgh, with a Sheraton bureau and book-case from the medical and nursing staff. Miss Sandford has held the position which she now vacates in consequence of indifferent health for ten years.

Tonbridge Union Infirmary.—Miss Beatrice Morgan, in view of her approaching marriage, having resigned her position as nurse at Tonbridge Union Infirmary, has been presented by the nursing staff with a handsome silver tea set. Dr. Crawford, in making the presentation, congratulated Miss Morgan, wishing her many years of happiness. Amongst other presents was a very handsome butter-dish from the master and matron.

### "The Hospital" Convalescent Fund.

The Hon. Sec. acknowledges with thanks the receipt of 10s. from "A. T.," and 2s. 6d. per The Hospital Travel Correspondent.

### Echoes from the Outside World.

### The Movements of Royalty.

The King held an investiture at Buckingham Palace on Friday last, and handed to about 150 recipients of birthday honours the insignia of the various distinctions conferred on them. The decoration of the Royal Victorian Order was bestowed in 26 cases; Mrs. Mary E. Bruce, the only lady invested, received the decoration of the Royal Red Cross. It is notified that the King and Queen will hold a series of evening Courts at Buckingham Palace during the coming season, at which presentations of ladies to their Majesties will be made.

### Royal Christmas Presents.

The King and Queen were very busy during last week choosing Christmas presents. The Queen endeavours, as far as possible on such occasions, to select articles which have been made in England. Amongst other specialities delivered at Buckingham Palace for Royal inspection were some beautiful hand-painted boxes containing a large and varied selection of sweets, arranged, not in the usual layers but in a set pattern, the initials of the recipient, and sometimes also those of the giver, being worked in in silver dragees. These sweets, as well as the boxes themselves, were made in England, and are said to be quite as good as the Parisian confectionery. The King is particularly fond of presenting books in beautiful bindings as Christmas gifts, and he sometimes gives special orders regarding a favourite volume of poetry or prose which he wishes to have bound in some special style. Of course a large number of toys are sent to the Palace for the Royal grandchildren, and the Queen is also careful to remember the servants, especially those who have been a long time in her Majesty's service.

### The Cumberland Silver Wedding.

The Duke and Duchess of Cumberland celebrated their silver wedding on Sunday, the betrothal of their second daughter, Princess Alexandra of Cumberland, to the Grand Duke Frederick Francis of Mecklenburg-Schwerin taking place at the same time. King Christian of Denmark and his son, Prince Waldemar, came from Copenhagen in order to present his congratulations in person, not only to his daughter, Princess Thyra, who was married to the Duke of Cumberland in 1878, but also to his granddaughter, Princess Alexandra, whose elder sister, Princess Marie Louise, was married to Prince Maxmillian of Baden in 1900. On this occasion of double interest the town of Gmunden was illuminated, and the Duke presented 35,000 kronen to local charitable institutions.

### Royal Christening of a Battleship.

On Saturday afternoon the Duke and Duchess of Connaught attended the launch of the first-class battleship *Hindnstan* at Clydebank, Glasgow, the christening ceremony being performed by the Duchess. In spite of drizzling rain, a large concourse of people assembled to witness the launch, and at the conclusion of the short religious service the Duchess of Connaught stepped forward and dashed a bottle of wine against the ram of the ship, at the same time naming her the *Hindustan*. At the luncheon which followed the ceremony, the Duke of Connaught, acknowledging the gift to the Duchess of a gilt casket containing the scissors with which she cut the ribbon that released the battleship, said that she was very pleased to have been authorised by the King, at the request of the Admiralty, to associate her name with the vessel. He hoped that the christening of the ship would knit closer that bond of union and loyalty which existed between the Indian Empire and the Mother Country.

### The Duke of Norfolk's Engagement.

It is announced that the Duke of Norfolk is engaged to be married to the Honourable Gwendolen Mary Constable-Maxwell, elder daughter of Lord Herries, and heiress to the ancient Scotch barony of her father, the eleventh peer. The Duke is premier Duke and Earl in the English peerage, and besides being Hereditary Earl Marshal is Chief Butler of England. He is 56 years old, and Miss Constable-Maxwell was born in January, 1877. She is, of course, a Roman Catholic. The first wife of his Grace, who was Lady Flora Hastings, died in 1887, and his only son last year.

### An Object Lesson in Discipline.

That twice within a month a large number of young children should have been saved from a burning school building without loss of life or injury to a single being, speaks well for the presence of mind of the teachers and for the excellent discipline maintained. A few weeks ago 2,000 children in East Ham were marched quietly out of the school house directly the flames were discovered, and last Friday 1,200 scholars had a similar experience at Glasgow. As soon as the head master of the school had been told that a fire had originated in the cooking-room, he sent someone to turn off the gas at the meter, and then visited the girls' and boy's quarters and made them file out into the streets in regular order. Realising that the little ones might be frightened by the sounds of shouting made by those around, when they saw smoke issuing from the building, he bade one of the pupil teachers sit down to the piano and all the 350 infants passed into the open air away from danger, stepping out bravely to "Napoleon's Grand March." As soon as the rooms had apparently been cleared, the head master instructed his male assistants to search every corner of the building so that no one could have been overlooked. The damage is estimated at from £2,000 to £3,000.

### Death of Clifford Harrison.

The death was announced last week, at Hastings, of Mr. Clifford Harrison, the accomplished reciter, and the burial took place on Monday. Mr. Harrison was a victim of consumption, from which he had suffered for some time. He was not unprepared for the end, and last summer gave a farewell series of recitals at Steinway Hall. Clifford Harrison had enthusiastic admirers in many circles, and had recited frequently before the King and Queen when they were Prince and Princess of Wales. It sometimes took him months to study the elocution of a piece and its musical accompaniment. The member of a family of actors, he was at first on the dramatic stage himself, but found his vocation as a reciter. Clifford Harrison was brother-in-law to "Lucas Malet," Charles Kingsley's daughter, and for years included Kingsley, Ruskin, and Browning among his friends.

### Miss Corelli's Libel Suit.

Last week Miss Marie Corelli brought an action against a Stratford-on-Avon newspaper for libel. Two hours before the case came on, the court at Birmingham was invaded by persons who were only admitted with special tickets issued by the Under-Sheriff, so great had been the desire to see the novelist in the witness-box. The alleged libel was the insinuation that Miss Corelli would not have opposed the erection of a free library in Henley Street, Stratford-on-Avon, if it had been associated with her name instead of with that of Mr. Andrew Carnegie. The plaintiff, in her evidence, denied that she had any intention of presenting a library to the town, and affirmed that her only object in opposing the Carnegie scheme was to prevent the destruction of certain cottages which she regarded as Shakespearian relics. In the result the jury gave her a verdict, but with only a farthing damages, and each side has therefore to pay its own costs.

### A Book and its Story.

NEW NOVEL BY THE AUTHOR OF "SHIPS THAT PASS IN THE NIGHT."*

Miss Beatrice Harraden has written more than one book since "Ships that Pass in the Night," but it is by the pathetic story, so valued by readers who can appreciate its rare charm that she is, perhaps, best known. The heroine's character belongs to a type that is perennially interesting, and it is one which, in its strength and sweetness, has fundamentally many points in common with that of her new heroine. Katherine Frensham is essentially feminine. With a magnetic charm that is a source of attraction to everyone coming within its influence, she yet remains, while winning the devotion of the opposite sex, heart-whole herself until long past youth.

She first comes into the story on the eve of her brother's wedding. "She and Ronald had always been close friends, and their companionship had ever been a joy to themselves and all who knew them. Since childhood they had been called 'the inseparables.'" Together they had loved their mother passionately, and when she died she said to them, "Love each other always, promise me, whatever comes, whatever befalls, stand by each other." They grew up, made their home to gether alone. Ronald became head of the organ-building business left to him and Katherine by their father, and thus they were partners in business as well as in pleasure. . . . "Kath, dear old senior partner, I feel terribly upset about you—now it comes to the point—I— " here he broke off, but there was no need to finish the sentence, for Katherine knew. "It is all right, dear old chap," she answered, "and, you see, we are friends for life. And I might have been the one to leave you. I nearly did three times!" "Four," he said, quaintly, "you never own up to four times!" And they both laughed. They had had many merry times over some of Katherine's passing love affairs. "At least you will live near us," Ronald said. She shook her head. "I am going to travel. I am going to the ends of the earth. I have always wanted to see the great countries of the world, and this is my chance. You have someone to love you and to look after you and I can go forth. Don't give up your music Don't give up your Wednesday quartette meetings."

After three years' wandering Katherine returned to England. To take up the broken thread of her life under its new conditions was at first difficult and sad. "Why did I return? If there was nothing and no one to return for, why should I have returned? Home sickness—ah, yes—and the love of the old country. But even then, if one has no ties, and is not wanted, what is it all worth? One country is as good as another if there is no love-niche anywhere, and there can be no loneliness greater than that found in old conditions changed to new." Katherine, standing alone, face to face with the future, looked "like some strong tree left standing on the mountain side to face the tempest alone. She was tall, made on a grand scale. As there was nothing petty in her attractive appearance, so also there was nothing petty in her mind.

With the gratitude of a simple nature thankful for the joy of happy memories, she consoled herself with the reflection that "No person on earth has a right to grumble who has had thirty-six years of close companionship with some beloved one. And it was a splendid time, something to give thanks for all the rest of one's life."

The evening of her return happened to be one on which she found Ronald alone during the temporary absence of his wife. Her sister-in-law and she had nothing in common. "They spoke a different language," was the only criticism Katherine expressed to an old friend. On this evening of reunion with her brother she found much to talk over; old scenes, old difficulties, old memories of the happy past.

Ronald shared with Katherine a love for music, and they had gathered round them a little band of devoted musicians who formed a quartette party and met at his house. It was of these musical evenings she was thinking. when she had begged him not to give up his music. He turned to her after speaking of the three musicians, Katherine's devoted friends and adorers, Signor Luigi, Herr Edelhart, Mons. Gervais, and said, "Do you know to-night is the last of our quartette meetings. Gwendolen does not like them. They seem to interfere with her arrangements." Katherine was silent. Later on, when the musicians arrive and everybody offers their homage to Katherine, coupled with expressions of delight at her presence among them again, her brother reminds her that she has come back to her faithful admirers. "Faithful flatterers," Katherine answered, as she stood in their midst shaking hands with them repeatedly. The little Frenchman was radiant with delight, and exclaimed "Mon Dieu! Mademoiselle have returned to us. Ah! le climat detestable of England have become a beautiful French printemps. The fogs is gone. My dead heart is alive, and Mademoiselle have made the miracle!" Ronald announces that he expects a friend, a stranger to them all, who is coming to listen to the music. "A stranger," they cried, "and on our last night." "Who is this stranger," Katherine asked, "and how dare he intrude at such a moment?" Ronald explained that he was a neighbour of his, during a stay in Surrey, who had lost his wife under tragic circumstances. "He is a sad man and we must not let our gaiety jar upon him.' When the stranger, announced as "Professor Clifford,' entered the room, Katherine, who had risen to receive him,' was startled by his remark, "But surely I know you?" and "I know you, surely." "Where have you met?" said Ronald. "I do not know," they said together, and they still stood motionless, arrested both in body and spirit. A little later in the evening we find Clifford Thornton and Katherine "sitting apart lost in the wonderful regions which music opens to everyone desirous of entering." Clifford Thornton entered and found the paths of peace. "Ah! how one rests," thought the man. "Ah! what an aimless, lonely life I've been leading," thought the woman. In the meeting of Katherine and Clifford centres the real interest of the book. The characterisation in Katherine Frensham is excellent, and we regret that space does not admit of further notice of many other admirably delineated characters. The Norwegian scenes make a delightful interlude between the first and latter half of the story. The following lines preface "Katherine Frensham," and at once place the reader *en rapport* with its intention.

"Midway the road of our life's turn they met,
And one another knew without surprise;
Nor cared that beauty stood in mutual eyes;
Nor at their tardy meeting nursed regret.

To them it was revealed how they had found
The kindred nature and the needed mind,
The mate by long conspiracy designed;
The flower to plant in sanctuary ground."

*G. Meredith.*

* "Katherine Frensham." By Beatrice Harraden. (Blackwood and Sons. 6s.)

### Notes and Queries.

**FOR REGULATIONS SEE PAGE 135.**

*Children's Nurse.*

With reference to the answer to *A. H. G.* (No. 99) last week, we are informed that the Home at Uplands, Loughton, has been transferred and is now being carried on as a home for children and training school for lady nurses at Court Royal, South Norwood Hill, S.E.

*Hospital Training.*

(113) Will you kindly inform me if mental nurses are accepted as probationers in general nursing at a London hospital? I have been told that the matrons of London hospitals object to them.—*B. P.*

It is quite true that many matrons prefer to train their own probationers from the beginning, but it is quite possible for mental nurses, who are otherwise eligible, to obtain general training in a public training school. "The Nursing Profession: How and Where to Train" (Scientific Press), may enable you to judge which institution best meets your needs.

*Abroad.*

(114) Will you kindly give me the address of one or more hospitals in Cairo or Rome where an English fully-trained nurse would be likely to find a vacancy? Perhaps you know of an agency in London which would be of service to me in furnishing addresses of hospitals on the Continent.—*Daisy.*

Write to the Matron of the Station Hospital, and the Kasr-el-Ainy Hospital, Cairo, for information. There is an English hospital in Rome. There is no association or agency for sending nurses to the Cont nent in London.

Will you kindly tell me if, after two years' fever training, it would be an easy matter to obtain an appointment in America or Australia? Is there a paper like The Hospital published weekly in both continents, and where can I get copies of them?—*E. A. T.*

It would be very difficult to obtain an appointment anywhere with only fever training. The "Trained Nurse" is the chief American nursing paper; it is issued monthly, and you can order it through the Scientific Press.

*Colonial Nursing Association.*

(115) Will you kindly tell me to whom to apply concerning the Colonial Nursing Association?—*M. A. B.*

Apply to the Secretary of the Association, Imperial Institute, S.W.

*Queen Alexandra's Nurses.*

(116) We should be glad if the Editor would kindly let us know where we could get information regarding Queen Alexandra's Nurses.—*Sister* and *H. P. B.*

Apply, The Matron-in Chief, Queen Alexandra's Imperial Military Nursing Service, Victoria Street, S.W.

*Hospital for Speech.*

(117) I shall feel greatly obliged if you can give me the address in London of the hospital for affections of speech, and the hours of attendance.—*L. C.*

Your query is too vague; please write more fully.

*Who Owns It?*

(118) I have been district nurse here for 12 months, and having two spinal cases—they are very poor—I have collected money when off duty to buy a spinal carriage for their alternate use. I hear that my committee claim the carriage, but, as they have not contributed towards its cost, I should like to know to whom it really belongs, and what you would do in the case?—*Nurse F.*

The carriage belongs to the subscribers. Ascertain for certain whether the committee claim it; if they do, ask the contributors to whom they wish it to belong.

**Important Nursing Textbooks.**

"The Nursing Profession: How and where to Train." 2s. net; 2s. 4d. post free.

"A Handbook for Nurses." By Dr. J. K. Watson. 5s. net; 5s. 4d. post free.

"Practical Guide to Surgical Bandaging and Dressings." By Wm. Johnson Smith, F.R.C.S. 2s. post free.

"The Nurses' Dictionary of Medical Terms and Nursing Treatment." By Honnor Morten. 2s. post free.

"The Human Body: its Personal Hygiene and Practical Physiology." By B. P. Colton. 5s. post free.

"Art of Feeding the Invalid." (Popular Edition). 1s. 6d. post free.

"On Preparation for Operation in Private Houses. By Stanmore Bishop, F.R.C.S. 6d. post free

### For Reading to the Sick.

FAITH.

Faith is the dawning of day
Where darkness was before,
The rising of a solar ray
To set in night no more.

Faith is the prop on which we lean
In darkness or distress,
Far oftener felt and known, than seen
Throughout this wilderness.

Faith yields a sense of life and love
Upborne on wings of prayer,
Swift as an eagle or a dove
That cleaves the liquid air.

*M. Bridges.*

And if at some dark hours our hearts sink, and we wonder whether anything is being achieved, whether our hope can be real, whether it can be worth while to wait on and trust, then, beloved, let us remind ourselves that we have no gauge by which to measure the gains and the losses. We are not in a position to estimate God's winnings, for we know not yet what we shall be hereafter; we know not what God has in view, in store. His ultimate aim is hidden, far, far beyond the veil of death. And in view of that hereafter He may well be gaining more than we think out of this dark and chaotic probation on earth. For God gains if only he can save a soul from that deliberate and defiant recoil from holiness which makes the case desperate. He gains if only he can secure in a soul that its deepest wish, its core of will below all its wobegone falls, have something in it of belief in goodness, of appeal to God.—*Canon Scott Holland.*

Dear friends, if we cannot fall down before God to-day, let us bow our hearts the more; let us tell Him that we are trying to take our illness or our pain patiently, to show Him that we are sorry from our hearts for having ever offended Him. Let us tell him "all the truth;" then, even if our prayers are sent up from sick beds of pain and weariness, our Lord will feel our trembling touch, He will know our longing heart, and will say to us, "Be of good comfort; thy faith hath made thee whole; go in peace."—*B. Waugh.*

Everything which happans to us comes from Thee, O God. It is Thou who hast done it for our eternal welfare. In the light of Eternity we shall see that what we desired would have been fatal to us, and that what we would have avoided was essential to our well-being; it is Thou who doest all things; it is Thou who during every moment of our lives art the life of our hearts, the light of our eyes, the soul of our souls.—*Fénélon.*

Methinks if ye would know
How visitations of calamity
Affect the pious soul, 'tis shown you here.
Look yonder at that cloud, which, through the sky
Sailing along, doth cross in her career
The rolling moon. I watched it as it came,
And deemed the deep opaque would blot her beams;
But, melting like a wreath of snow, it hangs
In folds of wavy silver round, and clothes
The orb with richer beauties than her own;
Then, passing, leaves her in her light serene.

*Southey.*

## Figures and Tables

**Figure f1:**
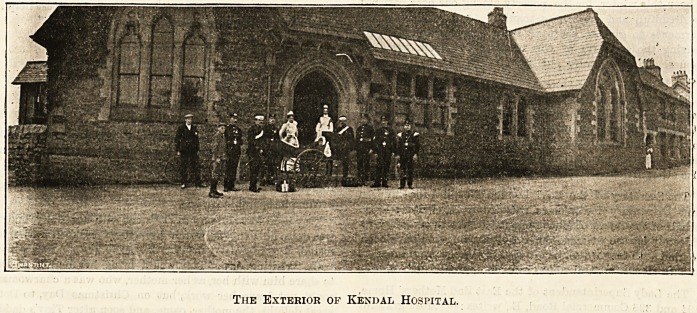


**Figure f2:**
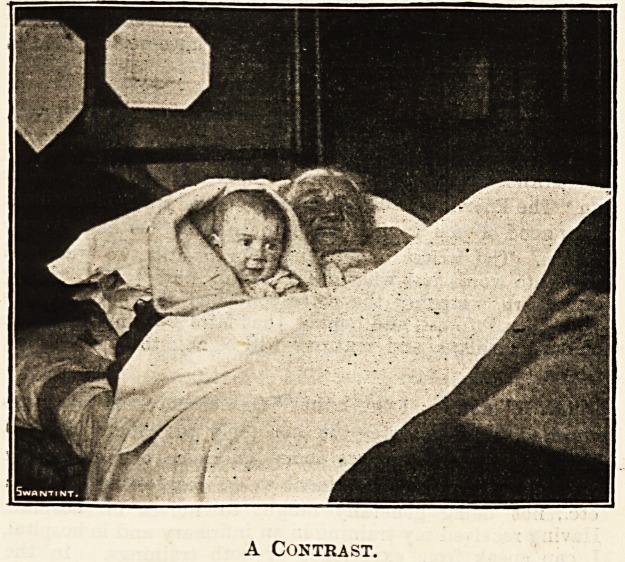


**Figure f3:**